# Application of 3D modeling and fusion technology of medical image data in image teaching

**DOI:** 10.1186/s12909-021-02620-z

**Published:** 2021-04-06

**Authors:** Quan Yuan, Xiaomei Chen, Jian Zhai, Yadi Chen, Qingxiang Liu, Zhongxiao Tan, Gao Chen, Kangle Zhuang, Jianying Zhang, Xi Xu, Di Qiang, Xuefei Shao

**Affiliations:** 1 Department of Imaging, Yi-Ji Shan Hospital, Wannan Medical College, Zheshan West Road on the 2nd, Wuhu, 241000 Anhui China; 2 Hewanlan digital S T CO.LTD, Shuihu Road on Room204 First building, HeFei, 230000 Anhui China

**Keywords:** 3D modeling, PACS, Medical image;image teaching

## Abstract

**Background:**

We combined anatomy with imaging, transformed the 2D information of various imaging techniques into 3D information, and form the assessment system of real medical imaging cases in order to make up for the deficiencies in the current teaching of the medical imaging technology students.

**Methods:**

A total of 460 medical imaging students were selected and randomly divided into two groups. The research group received the teaching of the fusion of the original CT and MR data 3D model and the original image combined with 3D anatomical image. CT and MRI data are imported through load DICOM of 3D slicer. Different tissues and organs are segmented by threshold and watershed algorithm of segment editor module. Models are exported through export / import models and label maps in segmentation. Save the NHDR file of the original data and Obj file of the corresponding model through save the NHDR and corresponding Obj files are loaded into probe 1.0 software. The software can give different colors to the three-dimensional models of different organs or tissues to display the stereo models and related data, and display the hook edges of organ models on coronal, sagittal and axial images. At the same time, annotation can be established in the corresponding anatomical position. Finally, it can be saved as a single file of Hwl, and the teaching can be opened at any time through the program of probe 1.0. Statistical analysis Academic self-efficacy scale and Self-directed learning ability scale was adopted by self-directed learning evaluation scale between two groups.

**Results:**

Compare the theoretical scores and case analysis scores of the two groups. The scores of the study and control groups were significantly higher than those of the control group. Before the experiment, no significant difference was detected in the self-efficacy of learning ability and learning behavior between the two groups, while after the experiment, these differences between the two groups were statistically significan. Moreover, the learning ability self-efficacy and learning behavior of the two groups of students after the experiment was significantly higher than that before the experiment. The self-efficacy of the learning behavior of the control group was higher after the experiment than that before the experiment, albeit the difference was not statistically significant.

**Conclusions:**

The modern, information-based and humanized experimental teaching mode will be constantly improved under the support of PACS system in order to optimize the medical imaging teaching activities for the development of modern medical education.

## Background

Imaging is an applied technology formed by the intersection of many subjects. In recent years, Computed Tomography(CT), Digital Radiography(Dr), magnetic resonance imaging(MRI), single photon emission computed tomography (SPECT), and other imaging technologies have matured gradually and used widely in the diagnosis of medical diseases.However, the training of medical imaging students is still lagging, which could be attributed to the reason that anatomical knowledge cannot correspond to the imaging findings, the two-dimensional (2D) information in each imaging examination cannot be 3D, and the different imaging manifestations of real cases in different stages of the disease cannot be understood clearly. Therefore, we combined anatomy with imaging, transformed the 2D information of various imaging techniques into 3D information, and form the assessment system of real medical imaging cases in order to make up for the deficiencies in the current teaching of the medical imaging technology students [1]. Therefore, 460 students majoring in medical imaging at Wannan Medical College were selected to analyze the advantages and disadvantages of the traditional teaching model and the current teaching model.

## Methods

### Student information

A total of 460 medical imaging students who studied sectional anatomy in our College from September 2017 to October 2018 were selected and randomly divided into two groups. A total of 228 students who adopted the traditional teaching model, included 102 boys and 126 girls, aged 19–25 (average: 22.9 ± 1.0) years. A total of 232 students (109 boys and 123 girls), aged 20–27 (average, 22.7 ± 1.0 years), were enrolled in this study. Moreover, no significant difference was detected in the gender and age between the two groups.

### Design

**Fig. 1:**
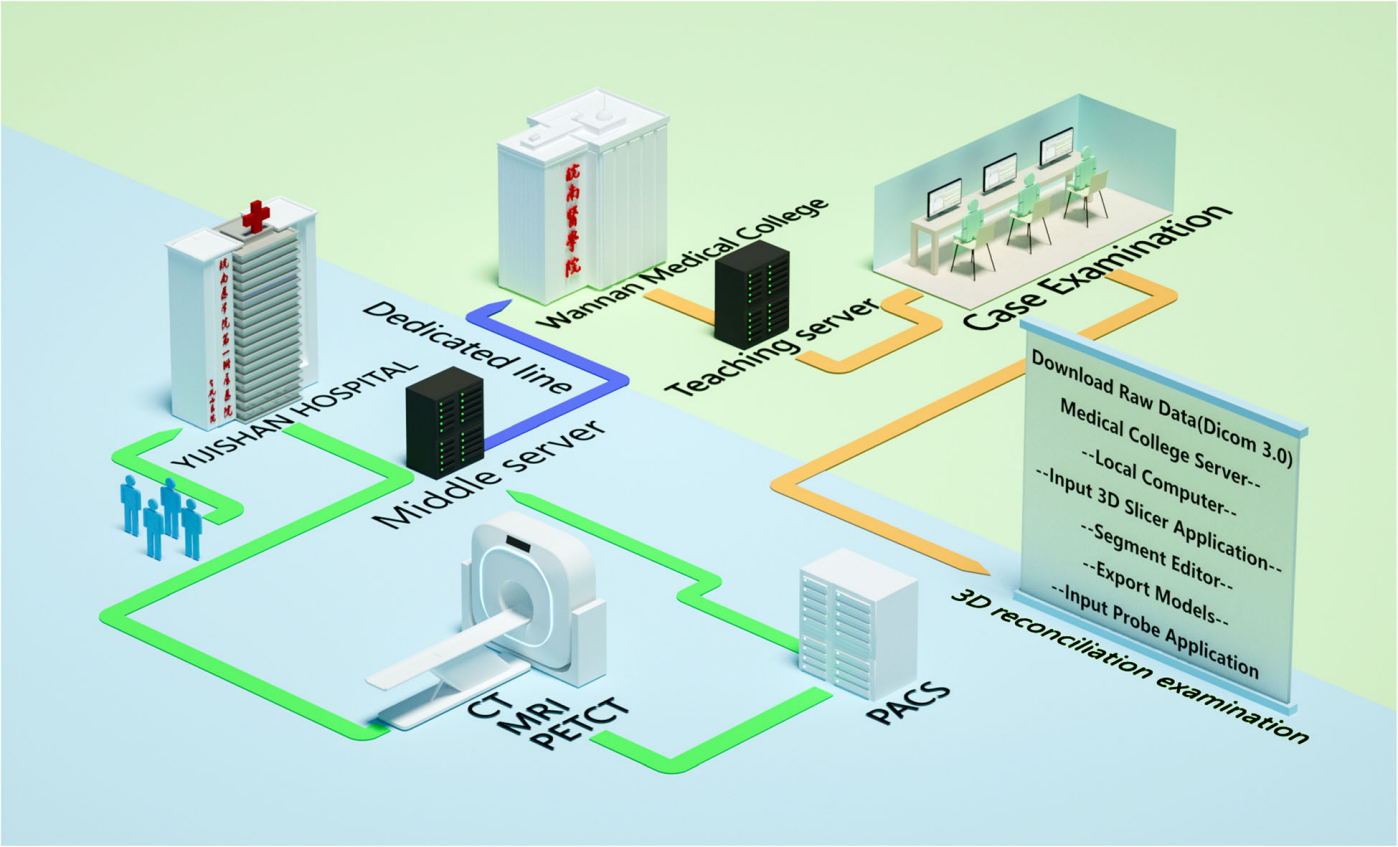
Experimental flowchart

The control group received traditional teaching model, including traditional experimental teaching and course teaching. The research group received the teaching of the fusion of the original CT and MR data 3D model and the original image combined with 3D anatomical image. CT and MRI data are imported through load DICOM of 3D slicer. Different tissues and organs are segmented by threshold and watershed algorithm of segment editor module. Models are exported through export / import models and label maps in segmentation. Save the NHDR file of the original data and Obj file of the corresponding model through save the NHDR and corresponding Obj files are loaded into probe 1.0 software. The software can give different colors to the three-dimensional models of different organs or tissues to display the stereo models and related data, and display the hook edges of organ models on coronal, sagittal and axial images. At the same time, annotation can be established in the corresponding anatomical position. Finally, it can be saved as a single file of Hwl, and the teaching can be opened at any time through the program of probe 1.0. All the teachers are attending doctors for > 5 years with abundant practical and teaching experiences. The experimental flowchart is illustrated in Fig. 1.

### Teaching implementation

#### Research group

**Fig. 2:**
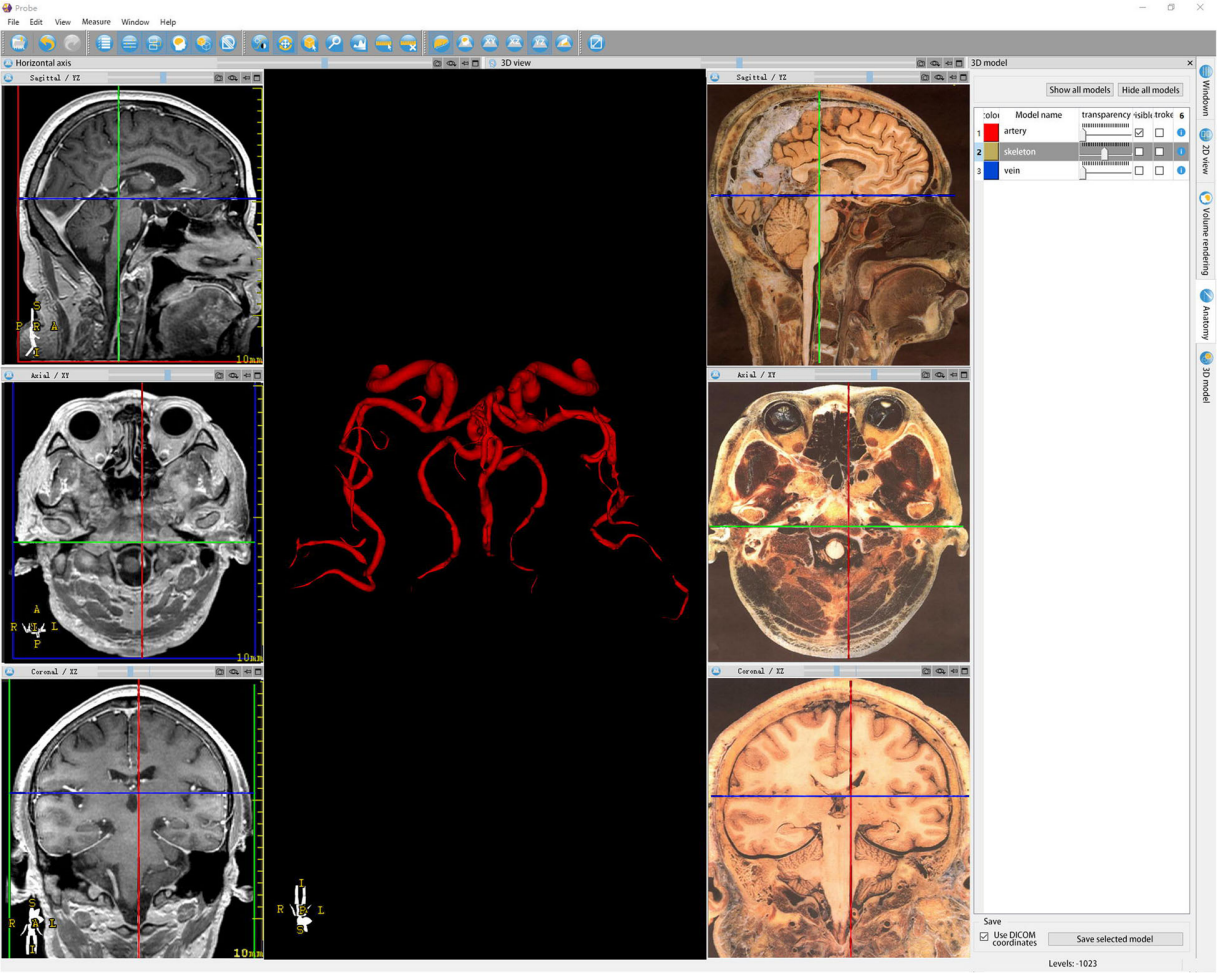
Cranial MRI and corresponding sectional anatomy. The left side is the head MRI, the right side is the corresponding sectional anatomy, and the middle panel is the three-dimensional cerebral vascular imaging reconstructed by MRI, which can be rotated and magnified arbitrarily

**Fig. 3:**
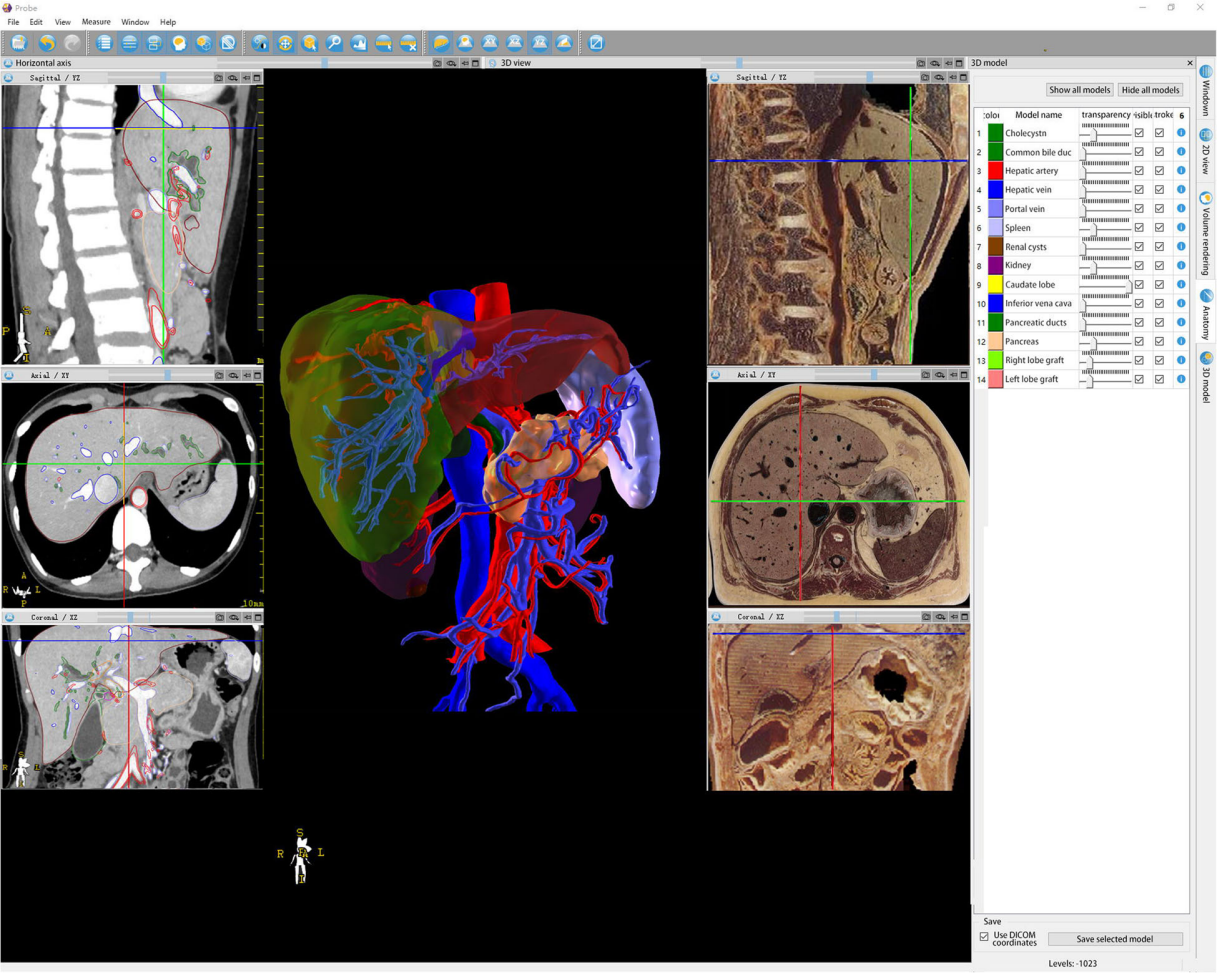
Liver CT and corresponding sectional anatomy. The left side is the liver CT, the right side is the corresponding sectional anatomy, and the middle panel is the 3D liver and blood vessels reconstructed by CT. It can be rotated and magnified arbitrarily

1. The corresponding three-dimensional model was generated using the existing images of the head, neck, chest, abdomen, male and female pelvic cross-section specimens, and the corresponding images of the above parts. These data were imported into Probe 1.0 software. (Figs. 2, 3).
2. The 3D dynamic model of each important organ was fused with CT and MR images, including the voxel reconstruction model and surface rendering reconstruction model.

#### Control group

Systematic anatomy, regional anatomy, surgery and medical imaging were utilized to give theoretical lectures to the students, explaining the anatomical structure of each system, clinical and imaging manifestations of common diseases, and diagnosis and differential diagnosis methods.

### Effectiveness assessment

#### Theoretical examination and case analysis examination

**Fig. 4:**
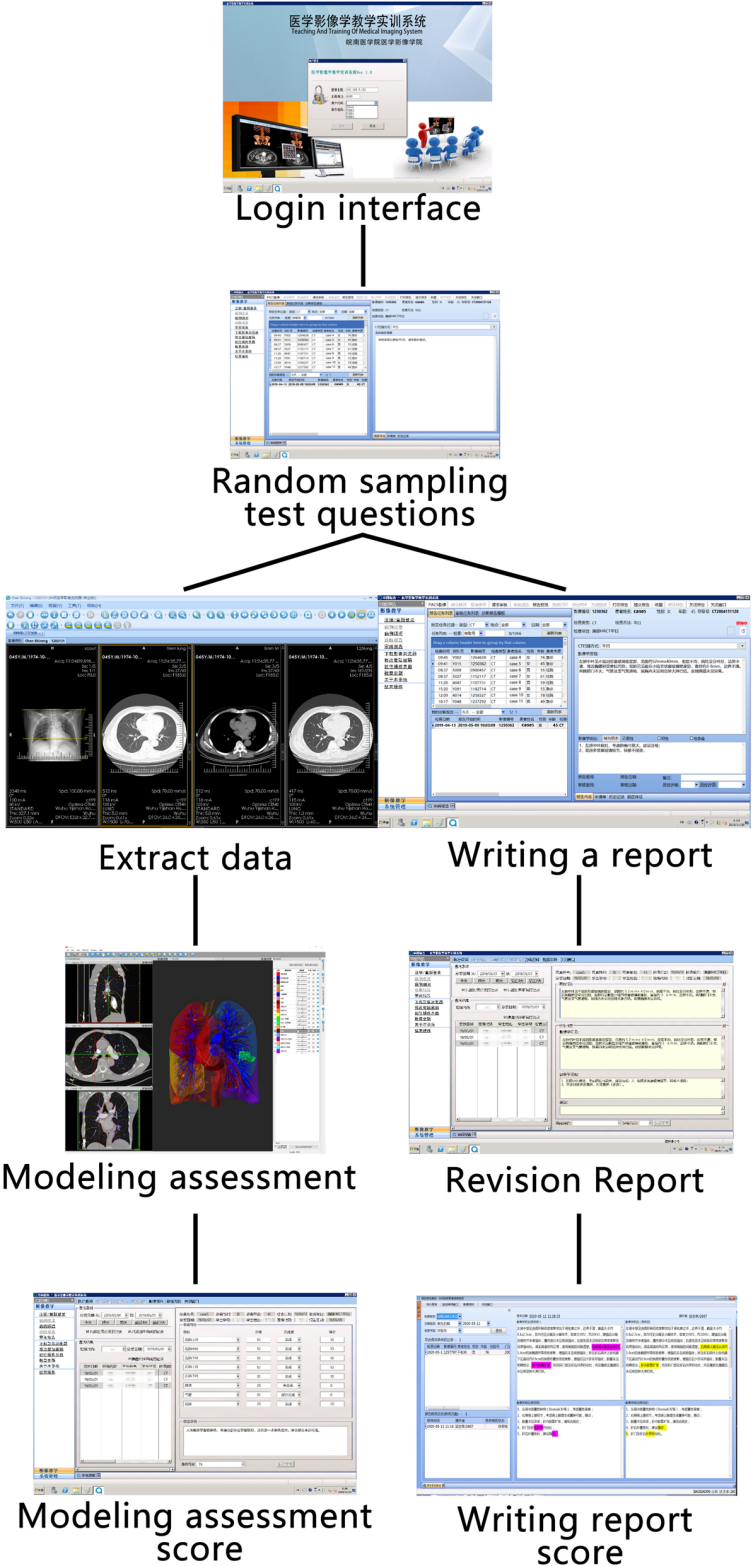
Student case assessment flowchart is divided into real case image report writing and data extraction to establish a three-dimensional model. Lessons for Practice. This teaching method is easier for students to accept and can let students contact clinical earlier

The full score is 100, and the same teacher will mark the paper. The case for analysis was selected randomly by students. These were real clinical cases in the Affiliated Hospital, including Dr., CT, and MRI. All students will take the following step test (Fig. 4).

### Academic self-efficacy scale

The academic self-efficacy scale of college students, including two dimensions, learning ability self-efficacy and learning behavior self-efficacy was assessed with respect to a total of 22 items A 5-level Likert scoring (1–5) method was applied: “completely inconsistent,” “relatively inconsistent,” “uncertain,” “relatively consistent,” and “fully consistent,” respectively. The stronger the students’ academic self-efficacy, the higher the score.

### Self-directed learning ability scale was adopted by self-directed learning evaluation scale (SRSSDL-CV)

The tool includes learning awareness, learning behavior, learning strategy, learning evaluation, and interpersonal skills, with 12 items in each parameter. The Likert 5-grade scoring method is used to score 1–5 for “never”, “rarely”, “sometimes”, “often”, and “always”. The stronger the self-directed learning ability, the higher the score [2].

### Statistical analysis

The measurement data were represented as $\overline{\mathrm{x}}\pm \mathrm{s}$.The comparison between the two groups was conducted by group t-test, and the self-comparison was conducted by paired t-test. The enumeration data were expressed by n (%) and the two-sided test. SPSS20.0 statistical software was used for data analysis. The test level was selected as α = 0.05.

## Results

### Comparison of the results of the two groups of students

T-test was used to compare the theoretical scores and case analysis scores of the two groups. The scores of the study and control groups were significantly higher than those of the control group (*P* <  0.05; Table 1).

**Table 1:** Comparison of theoretical and case analysis results of students ($\overline{\mathrm{x}}\pm \mathrm{s}$)

Project	Research group (***n*** = 232)	Control group (***n*** = 228)	***t***	***P***
Theoretical achievements	84.4 ± 6.5	73.0 ± 8.1	16.689	< 0.01
Case analysis results	84.7 ± 5.5	73.0 ± 8.4	16.671	< 0.01

### Comparison of self-efficacy between two groups of students

T-test was used to compare the academic self-efficacy between the two groups. Before the experiment, no significant difference was detected in the self-efficacy of learning ability and learning behavior between the two groups (*P* > 0.05), while after the experiment, these differences between the two groups were statistically significant (*P* <  0.05). Moreover, the learning ability self-efficacy and learning behavior of the two groups of students after the experiment was significantly higher than that before the experiment (*P* <  0.05). Moreover, the self-efficacy of the learning behavior of the control group was higher after the experiment than that before the experiment, albeit the difference was not statistically significant (*P* > 0.05). The total comparison of self-efficacy obtained the same conclusion (Table 2).

**Table 2:** Comparison of students’ self-efficacy ($\overline{\mathrm{x}}\pm \mathrm{s}$)

Groups	project	Self-efficacy of learning ability	Self-efficacy of learning behavior	Total
Research (n = 232)	Before experiment	31.1 ± 1.4	32.5 ± 1.0	63.5 ± 1.8
	After experiment	32.0 ± 0.9*^#^	33.1 ± 1.4*^#^	65.2 ± 1.7*^#^
Control (n = 228)	Before experiment	31.0 ± 1.3	32.4 ± 1.2	63.4 ± 1.7
	After experiment	31.3 ± 1.3*	32.6 ± 2.0	63.5 ± 1.5

*Compared to before the experiment, *P* < 0.05; ^#^compared to the control group, *P* < 0.05

### Comparison of self-directed learning ability between two groups of students

T-test was used to compare academic self-efficacy between the two groups. Before the experiment, no significant differences were detected in learning awareness, learning behavior, learning strategies, learning evaluation, and interpersonal skills between the two groups (*P* > 0.05). After the experiment, the differences in the factors of self-directed learning ability between the two groups were statistically significant (*P* <  0.05), and the factors of the two groups were significantly higher than those before the experiment (*P* < 0.05; (Table 3). The total comparison of self-directed learning ability arrived at the same conclusion (Table 3).

**Table 3:** **Comparison of students’ self-directed learning ability (**$\overline{\mathbf{x}}\pm \mathbf{s}$**)**

Groups	Project	Learning awareness	Learning behavior	Learning strategy	Learning evaluation	Interpersonal skills	Total
Research (*n* = 232)	Before experiment	38.5 ± 2.3	38.1 ± 2.0	38.1 ± 2.5	41.9 ± 2.8	34.1 ± 1.3	190.8 ± 14.2
	After experiment	41.4 ± 3.2*^#^	41.6 ± 2.9*^#^	41.2 ± 3.4*^#^	45.1 ± 3.6*^#^	38.0 ± 3.1*^#^	207.4 ± 9.1*^#^
Control (n = 228)	Before experiment	38.5 ± 1.0	38.2 ± 1.2	38.2 ± 1.3	41.6 ± 1.3	33.2 ± 1.0	189.6 ± 2.6
	After experiment	38.8 ± 1.0*	38.8 ± 0.8*	38.9 ± 0.8*	42.5 ± 1.1*	33.8 ± 1.7*	192.8 ± 2.7*

*Compared to self before the experiment *P* < 0.05; ^#^Compared with the control group *P* < 0.05

### Comparison of teaching effect evaluation between two groups of students

Chi-square test was used to evaluate the teaching effect(stimulate interest in learning, improve the mastery of theoretical knowledge, strengthen clinical thinking ability, display subjective initiative, enhance problem analysis and resolution capabilities) in the two groups. The results showed that the study group stimulates learning interest, improves the mastery of theoretical knowledge, enhances clinical thinking ability, and shows a subjective initiative rate higher than that of the control group, albeit with significant differences (*P* < 0.05). The rate of problem analysis and solving ability in the study group was higher than that in the control group, but not statistically significant (*P* > 0.05) (Table 4).

**Table 4:** **Comparison of teaching effect evaluation between two groups of students [**n (%)**]**

Groups	Stimulate interest in learning	Improve the mastery of theoretical knowledge	Strengthen clinical thinking ability	Display subjective initiative	Enhance problem analysis and resolution capabilities
Research (n = 232)	196 (84.5)	201 (86.6)	199 (85.8)	194 (83.6)	197 (84.9)
Control (n = 228)	171 (75.0)	183 (80.3)	167 (73.2)	171 (75.0)	182 (79.8)
**χ**^**2**^	6.411	3.388	11.104	5.215	2.053
**P**	0.011	0.066	0.001	0.022	0.152

## Discussion

The traditional teaching of imaging anatomy is to observe and explore the anatomical details directly from the shallow to the deep on the limited corpses, and then to teach by combining the sectional specimen images and image findings [3]. The disadvantages of this teaching mode are as follows: 1. Anatomically, it is difficult to establish a 3D spatial position of organs, and the specimens are limited and cannot be repeated. 2. The imaging of anatomy is different from the real presentation. The purpose of imaging anatomy enables students to understand and master each anatomical structure corresponding to the images displayed by various imaging examinations. In this traditional teaching process, it is difficult for sectional anatomy pictures to correspond to images, and hence, difficult to show several continuous transition changes in the image. Third, traditional teaching cannot guide the students to think in three dimensions. The study of image anatomy is a transformation process from 3D to the plane and then to 3D. In order to accurately understand the human organs and other organizational structures, we should identify these 2D images through the visual process, with accumulated anatomical and imaging technology knowledge, while attempting to generate a virtual 3D human body structure. This requires students to have the ability to transform a 2D plane into 3D space.

The purposes of using probe software in this study were as follows: first, combining sectional anatomy with various images for an accurate correspondence; second, importing real case imaging data to generate 3D images to present the complex structure. Next, we imported the existing sectional anatomical images into the software, then integrated them with CT and MRI, and marked them point-to-point, to intuitively present the anatomical structure and the corresponding imaging manifestations, which in turn, would facilitate the students’ understanding and memory. In the case of complex structures, such as liver, brain blood vessels, and lungs, and other teaching difficulties, the reconstruction of 3D model describes its structure and distribution. Although it is a virtual reconstruction and is derived from real cases, it is not only conducive to teachers but also to guide students. During the transformation of 2D images into 3D images, the characteristics of the data of each image could be elucidated that stimulated the learning enthusiasm. In traditional teaching, due to the complexity and obvious changes of the head blood vessels, students will have some difficulties in understanding the actual spatial structure of the head blood vessels. Consecutively, the changes in the blood vessels shown in the 2D image are difficult for students to understand and identify the structure. The established 3D cerebral vascular model was used for teaching and comparing the blood vessels in anatomy and sectional images, through rotation, movement, cutting, more vivid, and intuitive explanation in order to deepen the students’ understanding of the vascular course. Also, through the continuous dynamic changes, students can further deepen their understanding and master the structure and changes [4].

This teaching uses a set of 3D software based on the original image data modeling to display the 2D images of the crown, loss, axis, and arbitrary section, as well as the 3D model established by the corresponding 2D image. In the teaching process, combined with the 3D model, we can organize the edge display on the 2D image or overlay the 2D image on the 3D model, in order to make students understand the learning objectives of images. 3D visualization technology provides a new way to solve this problem [5–8]. The 3D visualization analysis software could be used to establish 3D images of various organs, such as brain, lung, liver, and spine. The size and shape of the tubes, the shape and distribution of the main ducts, the size and location of the lesions, and the spatial correlation with the main structures could be displayed. Through transparent processing, screening, hiding, and other operation methods, the shape and branch distribution of each structure could be displayed separately to help students understand the correlation between the anatomical structure of the organ canal and the lesions. The students could improve their ability to understand the 2D images by observing CT or MRI and combining them with 3D images, thus stimulating their initiative in learning, improving learning efficiency, and achieving a positive feedback effect [9]. 3D visualization software could also be used to carry out virtual surgery. The students could observe the pathological changes on the computer, judge which pipelines need to be disconnected and which pipelines need to be retained, and compare the same with the actual operation and postoperative review to deepen the understanding of surgery. Not only these advantages are incomparable with traditional teaching methods [10] but the results of this study also show that the performance of the 3D teaching group is significantly better than that of traditional teaching group with respect to film reading, identifying organizational structure during operation, stimulating students’ learning enthusiasm, and helping to improve learning efficiency. The current findings also showed that the theoretical scores and case analysis scores of the two groups were 84.4 ± 6.5 and 84.7 ± 5.5, respectively; the scores of the study group were significantly higher than those of the traditional teaching group (*P* < 0.01).

As a response to sickness, the symptoms and manifestations of the human body are constantly developing and changing; thus, different imaging characteristics are observed in various developmental stages of the disease. The appearance of the same kind of disease will lead to different human immune defense functions and metabolic changes, which will present different image features. Interestingly, abundant clinical practice is required to make a comprehensive and accurate diagnosis by connecting the normal image with disease images through dialectical thinking. However, the learning cycle of students in school is limited, and teaching resources are relatively scarce. In addition, medical ethics greatly reduces the opportunities for the students to use hospital equipment and patients for training and learning. As a result, students’ operation opportunities and skill training cannot be guaranteed, and the learning cycle is limited, which does not allow the students to experience the complete evolution process of various diseases, severely affecting the cultivation of their practical ability. The current changes in medical education include that study by Frenk et al. that started the problem-based teaching method, which further linked the basic subjects to the clinical subjects [10]. The recent teaching reform focuses on the cultivation of students’ competency-based learning. In June 2015, Germany formulated and released the “National medical undergraduate education ability learning objectives,” which defined the objectives and importance of students’ ability training. Weinert et al. pointed out that learning ability refers to the ability to have or learn to solve certain problems using specific existing technologies successfully in various situations. In this study, SRSSDL was introduced to evaluate the students’ self-efficacy, which was first used to evaluate the nursing students [11]. Cadorin et al. [12] applied this scale for the assessment of radiology technicians and achieved satisfactory results; hence, we used this scale to compare self-efficacy in each groups. Before the experiment, no significant differences were detected in learning awareness, learning behavior, learning strategies, learning evaluation, and interpersonal skills between the two groups (*P* > 0.05); however, after the experiment, the differences in the self-directed learning ability were statistically significant (*P* < 0.05) between the two groups, and the factors of the two groups were significantly higher than those before the experiment (*P* < 0.05).

Subsequently, this study introduced the medical imaging teaching training and assessment system. PACS collects, stores, and transmits all kinds of imaging data through computer and network technologies, and browse and conduct the post-processing of the images. It has been widely used at the Radiology Department [13] in 2016 and was applied in the Affiliated hospital to store and diagnose the images of the clinical cases. With the rapid development in recent years, PACS has expanded from simple image storage and communication between radiation imaging equipment to the interoperability of all imaging equipment in the hospital, forming an integrated PACS of the whole hospital. In 2017, the affiliated hospital and medical college established a multimedia network training room based on PACS through the connection between the campus network and the affiliated hospital. In the teaching process, the teachers of the Imaging College made full use of the image resources such as CT, MRI, and DR in the Affiliated Hospital, sort them out according to the system, specialty, and chapter, and compiled the medical imaging database. The images can be used repeatedly and updated regularly. Students can study independently on the computer terminal. The medical imaging database could be used for students to learn, establish a simple medical imaging diagnosis test question bank, conduct diagnostic film reading examination, set up a discussion area for difficult medical imaging diagnosis diseases, and continue education [ [14, 15].

In the first experimental class, the teacher will introduce the basic operation of PACS. In each of the subsequent class, the teacher will first use the multimedia system to introduce the objectives of the experiment and propose the problems to be solved in the experiment. Students can access relevant pictures and knowledge in the PACS system or Internet and simulate clinical imaging report writing [16]. Then, the teacher solves the students’ questions in the inspection and finally makes a summary. The original teaching mode has been changed, and the teaching effect has been improved. Such network teaching based on PACS provides conditions for teaching reform. The teachers ask questions before class, and the students consult materials and documents through the PACS system and the Internet to solve problems. Finally, a summary is prepared by the teachers [17].

In teaching, through a large number of actual cases of picture observation, typical clinical cases, and discussion of difficult cases, students can further deepen the understanding of the law of disease occurrence and development. Compared to the traditional teaching model of imaging diagnostics training course, the electronic film reading library teaching method saves time, space, manpower, and material resources. Students can directly access and store images on the Internet, which is conducive to understanding and reviewing the difficulties and key points in teaching and is conducive to students’ review, induction, and summary after class [18].

The advantages of this teaching method lie are the convenience of distance learning and the improvement of students’ self-study ability. In traditional teaching, we could not check the film which has been put into the film library after teaching. While the network teaching of PACS is connected with the campus network, students can log into the PACS system through the campus network in their spare time for autonomous learning. It provides a convenient way for students to preview before class and review after class, thereby improving their enthusiasm and ability of autonomous learning.

Digital acquisition, storage, management, transmission, and reproduction of image information is a new technology system with high technical content and practicality. It involves a wide range of aspects, including imaging medicine, digital image technology, computer-based communication, and software engineering. PACS system has been introduced in our hospital since 2010, which has shown strong advantages in clinical application. It also provides a new tool for the teaching of the medical imaging experiment course, and hence, is a good method to improve the level of imaging teaching experiment course and achieve a satisfactory teaching effect.

In addition to the correlation between medical imaging and the development of the disease, the focus is on the association between the development of imaging and the disease. With the widespread application of digital imaging equipment in modern medical imaging diagnosis, teaching and scientific research, medical imaging information technology is developing rapidly. Thus, in the teaching practice, we should integrate new knowledge and new trends of people and strengthen the students’ ability to acquire knowledge, use knowledge, think independently, analyze comprehensively, and find and solve problems. Taken together, the PACS system exerts marked changes to the teaching mode of medical imaging. The modern, information-based and humanized experimental teaching mode will be constantly improved under the support of PACS system in order to optimize the medical imaging teaching activities for the development of modern medical education.

## Data Availability

The datasets used and/or analyzed during the current study are available from the corresponding author on reasonable request.

## Abbreviations

CT: Computed Tomography
DR: Digital Radiography
MRI: Magnetic resonance imaging
PACS: Picture archiving and communication systems
SRSSDL-CV: Self-directed learning ability scale was adopted by self-directed learning evaluation scale
